# Nanoformulations of essential oils for the treatment of fungal diseases, with a focus on dermatophytes

**DOI:** 10.1007/s00203-026-04746-4

**Published:** 2026-03-18

**Authors:** Ghulam Muhayyudin Chattha, Yasmin Louise Ramos de Andrades Cappellari, Walter Orlando Beys-da-Silva, Luiza Abrahão Frank, Lucélia Santi

**Affiliations:** 1 Faculty of Pharmacy, Federal University of Rio Grande do Sul, Porto Alegre, Brazil; 2 Post-Graduation Program in Pharmaceutical Sciences, Federal University of Rio Grande do Sul, Porto Alegre, Brazil

**Keywords:** Antifungal agents, Antifungal drug resistance, Nanotechnology, Dermatomycoses, Dermatophytosis, Natural products

## Abstract

Fungal infections, particularly those caused by dermatophytes, affect over 25% of the world’s population and pose a significant public health risk to both humans and animals. Conventional antifungal treatments face substantial challenges like drug resistance, adverse effects, and require long-term systemic medications. Essential oils (EOs) have attracted attention for their antimicrobial properties; however, their therapeutic applications have been hampered by their low solubility and rapid degradation. Nanotechnology addresses these limitations by encapsulating EOs to enhance their durability, bioavailability, and antifungal properties. This review covers the potential EO-based nanoformulations for treating fungal infections, with a focus on dermatophytes. It also highlights how nanoencapsulation may help reduce drug resistance, toxicity, and adverse effects. However, there are some limitations. The effectiveness of treating fungal infection can vary depending on the EO’s composition, fungal species, and how the nanoformulation is formulated. Future research should emphasize standardizing formulation protocol and evaluating in vivo efficacy and safety through well-designed clinical trials. While EOs represent a promising approach, they are not a substitute for evidence-based antifungal treatment and must not be used without medical guidance.

**Graphical abstract:**

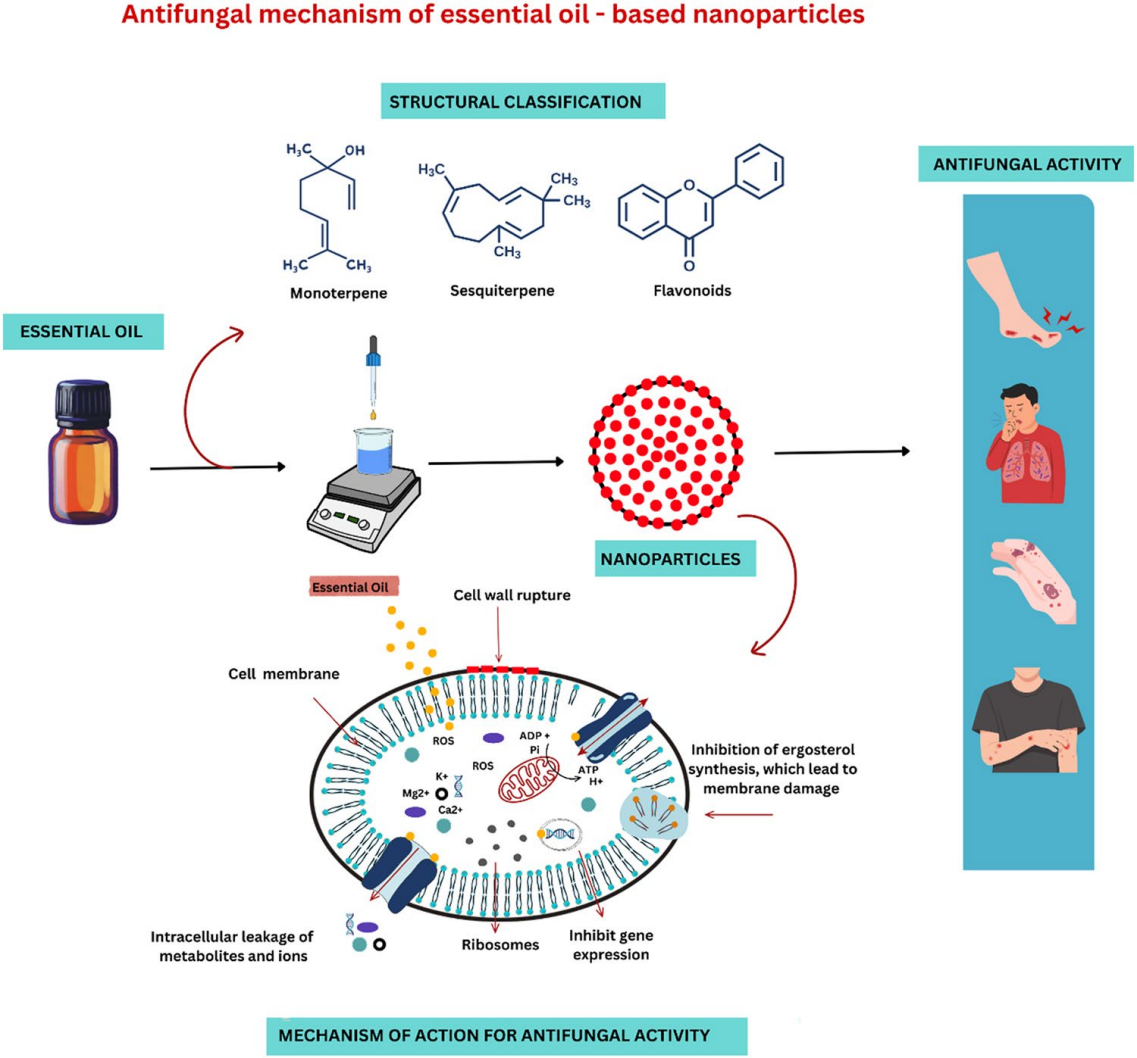

## Introduction

Dermatophytosis, commonly known as ringworm, is a prevalent infection in skin, nails, and hair caused by species from the genera *Trichophyton*, *Microsporum,* and *Epidermophyton* (Hainer 2003; Gupta And Cooper 2008). These fungi cause a mild to extremely severe infection by colonizing keratinized human or animal tissues. Dermatophytes are categorized into three ecological groups based on their primary host or habitat: geophilic, zoophilic, and anthropophilic. Geophilic dermatophytes typically dwell in soil and deteriorate keratinous substrates such as fur, feathers, hair, and horns (Hayette And Sacheli 2015). They are less prevalent than anthropophilic and zoophilic dermatophytes; geophilic species can occasionally infect humans, producing inflammatory skin lesions (Rad et al. 2018). Common geophilic dermatophytes are *Nannizzia fulva* and *Nannizzia gypsea* (formerly *Microsporum gypseum*).

Zoophilic dermatophytes primarily infect animals but can also be transmitted to humans through direct contact with animals or contaminated environments (Rad et al. 2018). These fungi frequently cause severe, inflammatory, and highly contagious infections that often require systemic and prolonged therapy (Baumbach et al. 2021). They are associated with various clinical presentations, including tinea manuum, tinea faciei, tinea pedis, tinea cruris, tinea corporis, and tinea capitis. Representative species include *Trichophyton verrucosum*, *Trichophyton mentagrophytes* var. *mentagrophytes* (granulosum), *Microsporum nanum*, and *Microsporum canis* (Spiewak And Szostak 2000). A recently identified species, *T. indotineae*, has emerged as a prominent pathogen in South Asia over the past few decades. It was previously classified as part of *T. mentagrophytes* but is now recognized as a separate species. *T. indotineae* is known for its high rate of genetic mutation in the squalene epoxidase (SQLE) gene, leading to resistance to terbinafine and complicating treatment (Uhrlaß et al. 2022; Jabet et al. 2023).

Anthropophilic dermatophytes are adapted to human skin and represent the leading cause of dermatophytosis worldwide (Gnat et al. 2020). This group includes approximately 10 species, the two major genera being *Trichophyton* and *Epidermophyton* (Hoog et al. 2017). *Trichophyton rubrum*, *Trichophyton interdigitale*, and *Epidermophyton floccosum* are the primary organisms responsible for most infections, with *T. rubrum* being the most common dermatophyte (Tang et al. 2024). Transmission occurs through direct contact from one individual to another or indirectly through fomites such as (such as upholstery, hairbrushes, and hats) (Kaufman et al. 2005).

In general, zoophilic and geophilic species cause more inflammatory disease, while anthrophilic species more commonly cause non-inflammatory infections (Shao et al. 2025). Table 1 outlines the various types of dermatophytosis, detailing the site of infection, prevalence, and common clinical manifestations associated with each type.

**Table 1:** Clinical and epidemiological characteristics of common dermatophytosis types, including infection sites, prevalence, and primary causative pathogens

Dermatophytosis type	Infection site	Prevalence	Clinical manifestations	Most common causative pathogens
Tinea corporis	Trunk and extremities	10–25%	Red, scaly rash with a raised border, often forming a ring shape	*T. rubrum, T. mentagrophytes, T. tonsurans* (Takenaka et al. 2020)
Tinea capitis	Scalp and head	20–25%	Hair loss, scaling, inflammation of the scalp, and round bald patches	*T. tonsurans, Mi. canis* (White et al. 2014)
Tinea pedis (Athlete's Foot)	Feet	10%	Itchy, red, peeling skin, often between toes or on soles	*T. rubrum, T. mentagrophytes, E. floccosum* (Hawkins And Smidt 2014; Ameen 2010)
Tinea cruris (Jock Itch)	Groin, pubic region	20–25%	Red, itchy rash in the groin, inner thighs, and buttocks, circular shape	*T. rubrum, T. mentagrophytes* (Gupta et al. 2003; Lee et al. 2015)
Tinea unguium	Nails	1–8%	Thickening, discoloration, and brittleness of nails	*T. rubrum, T. mentagrophytes* (Gupta et al. 2016; Ebihara et al. 2009)
Tinea manuum	Hands	10–25%	Dry, itchy skin, peeling, and redness on the hands	*T. rubrum* (Chamorro et al. 2025)
Tinea faciei	Face	20–25%	Red, scaly patches on the face, often with well-defined borders	*T. rubrum, T. mentagrophytes* (Pei et al. 2022)
Tinea barbae	Beard area	Uncommon	Red, itchy rash in the beard area, folliculitis-like appearance	*T. verrucosum, T. rubrum, T. mentagrophytes* (Kuruvella et al. 2025)

The prevalence of dermatophytosis varies significantly across regions due to differences in climate, socio-economic factors, hygiene practices, and the dominant fungal species. Globally, over 650 million people are affected by fungal skin infections. Dermatophyte infections, such as ringworm and tinea, account for more than half of these cases (Organization, W.H. Ringworm (tinea) 2025). In 2021, there were approximately 1.73 billion cases of fungal skin diseases, and prevalence is projected to increase through 2050 (Li et al. 2025). In Africa, prevalence rates among school-age children vary greatly, ranging from 10 to over 70%. The most common pathogens are *Trichophyton violaceum, Trichophyton soudanense,* and *Microsporum audounii* (Coulibaly et al. 2018). In Asia, dermatophytosis accounts for up to 70% of dermatological consultations, and the prevalence of *T. mentagrophytes* has increased from 20 to 90% over the past 15 years (Keshwania et al. 2023). In Brazil, the prevalence rate ranges from 18.2 to 23.2%, and high humidity and temperature significantly contribute to the disease burden. In comparison, developed regions such as Europe report lower prevalence rates, often below 5%, with *M. canis* and *T. rubrum* being the most commonly isolated species (Pires et al. 2014). Surveys conducted by the World Health Organization have revealed that dermatophytes affect approximately 25% of the world's population (Keshwania et al. 2023), making them a public health issue in several countries. In immunocompromised patients, invasive dermatophyte infections can be fatal (Kruithoff et al. 2023; Rayens And Norris 2022).

Mycology and nanotechnology have had a long history of mutual interaction (Mashitah et al. 2016). Nanotechnology has emerged as a promising strategy to improve the effectiveness of antifungal agents by reducing toxicity, preventing degradation, enhancing drug distribution, prolonging circulation time, and improving targeting (Souza And Amaral 2017). Furthermore, owing to their inherent antifungal properties, numerous metallic nanoparticles (MNPs) have been employed to combat plant and human pathogenic fungi (Mashitah et al. 2016), especially silver nanoparticles (Ag-NPs) (Noorbakhsh et al. 2011).

Nanoformulations of EOs have also gained attention as a potential antifungal strategy (Kapustová et al. 2021). Studies have shown that incorporating EOs into nanoformulation significantly enhances their antifungal activity against various fungal infections (Correa-Pacheco et al. 2018). Unfortunately, EOs are lipophilic substances that degrade easily under the effect of light, oxygen, moisture, and temperature. One effective solution to these challenges is nanoencapsulation, which protects EOs from degradation, increases their solubility, masks unpleasant odors or tastes, and enhances bioavailability (Gupta And Variyar 2016). Additionally, nanoformulations can provide controlled release, which will ultimately result in fewer adverse effects and increased effectiveness (Costoya et al. 2022).

Recent studies show a significant increase in research on the antimicrobial properties of EOs, since fungal infections affect over 25% of the global population. While earlier reviews have documented the general antifungal activity of EOs (Nazzaro et al. 2017; Swamy et al. 2016; Udourioh et al. 2025; Carson et al. 2006; Paul et al. 2020), a closer look reveals that most prior evaluations focus on broad applications or traditional delivery methods. However, there is a lack of up-to-date literature that integrates advanced nanotechnology into the treatment of dermatophytoses. Current reviews often lack a detailed examination of how nanoencapsulation addresses the inherent challenges of EOs, such as low solubility, rapid breakdown, and poor absorption, particularly for skin-resident fungal pathogens. Moreover, although some research has addressed combination therapies, it has not thoroughly examined the specific benefits of EO-based nanoformulations in reducing drug resistance and lowering systemic toxicity (Couvreur et al. 2002; Carson et al. 2006; Paul et al. 2020). To address this gap, this review offers a detailed examination of EO-based nanoformulations, focusing on their effects against dermatophytes, mechanisms of action, and the synergistic effect to enhance therapeutic efficacy while minimizing side effects. By highlighting the urgent need for alternative therapies to address conventional drug resistance, this research provides a fresh perspective on how nanotechnology can convert volatile EOs into stable, highly effective antifungal agents, laying the groundwork for future clinical applications yet to be explored in the existing literature.

## Common treatments for dermatophytosis

The choice of therapy for dermatophyte infections depends on the type of disease, location, severity, and associated microorganism. Topical treatment is generally recommended for superficial skin, nail, or hair infections (Jartarkar et al. 2021; Lanier et al. 2018). Common topical antifungal medications include polyenes (liposomal amphotericin B), allylamines (terbinafine, naftifine, and butenafine), and azoles (clotrimazole, miconazole, and oxiconazole). Topical agents are preferred as first-line therapy for uncomplicated superficial dermatomycoses due to their efficacy, ease of application, low cost, and minimal systemic adverse effects (Poojary 2017). However, topical formulation may not be as effective for extensive cutaneous disease or onychomycosis (nail infections), and overuse of topical antifungal agents can lead to the development of resistance (Gräser, et al. 2018).

Systemic therapy is indicated for severe, widespread, or recalcitrant infections; infections unresponsive to topical agents; and infections involving hair follicles or nails. Common systemic antifungals include itraconazole, terbinafine, griseofulvin, ketoconazole, and fluconazole. Oral agents achieve deeper penetration into skin and hair follicles, making them more effective for tinea capitis, onychomycosis, severe tinea corporis, and moccasin-type tinea pedis (AL-Khikani 2020).

Griseofulvin is the preferred treatment for tinea corporis, although treatment may extend from 12 to 24 months. Terbinafine is the second-best agent, with a shorter treatment duration, being successful for extensive cutaneous illness and onychomycosis, and achieving a higher mycological and clinical cure rate (87%). Still, its use may be limited by side effects such as headaches, gastrointestinal symptoms, and rash (Kaul et al. 2017; Frazier et al. 2021). Fluconazole has not been widely investigated in the treatment of onychomycosis, and the FDA doesn't approve it for this purpose; ketoconazole may cause hepatotoxicity (Khoza et al. 2017). Due to several side effects, long treatment durations, and ineffectiveness, especially for onychomycosis, the urgent need for new alternatives to treat dermatophytic infections is evident.

Furthermore, antifungal resistance may develop due to inadequate dosage or treatment duration (Hainer 2003). Resistance may be intrinsic or acquired. Intrinsic resistance refers to natural resistance to certain antifungal drugs or a family of antifungal agents that does not require mutation or the acquisition of additional genes. However, when a microorganism resists antifungal drugs, this is known as acquired resistance, often due to mutations or changes in fungal cell metabolism. Key mechanisms include drug efflux, biofilm formation, target enzyme modification, and mutation (Kadariswantiningsih et al. 2025). Table 2 lists the conventional antifungal drugs used to treat dermatophytes.

**Table 2:** Antidermatophytic activity of conventional antifungal drugs

Dermatophytes	Effective antifungal drugs and their MIC (μg/mL)						References
	Itraconazole	Ketoconazole	Fluconazole	Terbinafine	Griseofulvin	Voriconazole	
*Trichophyton rubrum*	0.30	0.75	1.04	0.07	0.53	0.39	Pashootan et al. (2022), Badiee et al. (2023)
*Trichophyton mentagrophytes*	24.7	17.7	0.07	0.20	2.35	–	Badiee et al. (2023), Khan et al. (2025)
*Trichophyton tonsurans*	0.02	–	0.73	0.08	–	–	Samaddar et al. (2025)
*Trichophyton Terrestre*	0.5	0.5	32	0.01	>8	0.06	Curatolo et al. (2021)
*Trichophyton interdigitale*	0.08	0.32	3.28	0.06	0.41	0.05	Shaw, et al. (2020)
*Trichophyton benhamiae*	0.05	0.56	–	0.02	0.76	0.05	Ansari et al. (2021)
*Trichophyton verrucosum*	1.75	32	64	0.002	4	0.62	Łagowski et al. (2020)
*Microsporum gypseum*	0.07	–	0.026	0.039	0.23	–	Katiraee et al. (2021)
*Microsporum canis*	0.06	0.12	5.39	0.04	–	0.04	Fernández-Torres et al. (2001)
*Microsporum audouinii*	1	–	64	0.06	4	0.5	Johansen et al. (2024)
*Epidermophyton flocossum*	20.035	0.43	10.44	0.093	2.31	0.174	Adimi et al. (2013)

Resistance to conventional antifungal drugs in dermatophyte infections shows significant regional variation from Brazil to Pakistan. In 2024, the first case of terbinafine-resistant *T. indotineae* in Brazil emerged, raising concern among local clinicians (Veasey et al. 2025). However, the failure rate of antifungal treatment is as high as 20–25% (Ferro et al. 2025). A study conducted in Pakistan reports resistance to fluconazole was 20% for *T. rubrum* and 36% for *T. mentagrophytes*, while resistance to itraconazole was 20% and 16%, and resistance to terbinafine was 16% and 26%, respectively. Moderate resistance was shown by *M. canis* and *E. floccosum*, with rates of up to 21% and 24% for fluconazole, respectively (Siddiqui et al. 2025). This data highlights the importance of investigating alternative therapies to treat dermatophytosis.

## Overview of essential oils and their antifungal properties

The rise of microorganisms resistant to synthetic antimicrobials underscores the need for alternative treatments, particularly plant-based substances like EOs (Ibrahim et al. 2021; Nazzaro et al. 2017). Due to their varied chemical compositions, EOs exhibit a wide range of antimicrobial properties (Swamy et al. 2016). In the past, people used EOs for medicine, beauty, and spiritual reasons. Ancient civilizations such as Mesopotamia (circa 3500 BC), Egypt, and China left traces of EOs' use in medicine and daily life (Udourioh et al. 2025). Over 700 substances, including cinnamon, ginger, myrrh, and sandalwood oil, have been used in Ayurvedic medicine due to their healing properties (Kubeczka 2020; Wilken et al. 2011). Their popularity increased in Europe during the Middle Ages, where EOs were used to treat infectious diseases, including the Bubonic Plague (Jyotirmayee And Mahalik 2022). With advances in chemistry in the nineteenth and twentieth centuries, interest in EO research expanded, contributing to its widespread use today.

**Fig. 1:**
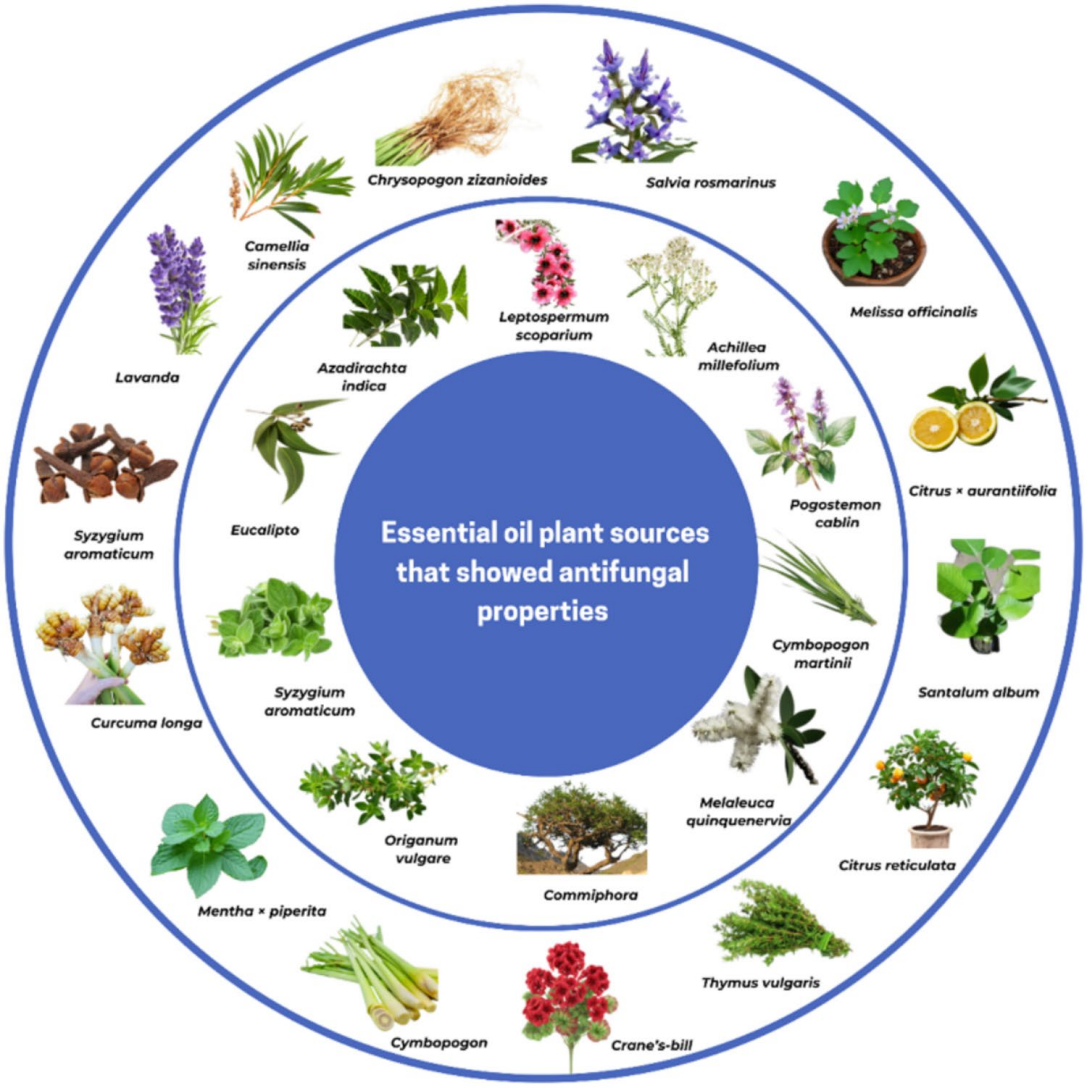
Overview of plant source essential oils with described antifungal activity

These days, EOs are used worldwide in aromatherapy, cosmetics, perfumes, and pharmaceuticals (Perveen et al. 2025). In plants, EOs function as defense agents by repelling pathogens and herbivores, while pollinators and seed dispersers are attracted (Sharmeen et al. 2021; Benelli et al. 2018). In plants, EOs function as defense agents by repelling pathogens and herbivores, while pollinators and seed dispersers are attracted (Dreger And Wielgus 2013; Barbieri And Borsotto 2018). Their antifungal, anti-inflammatory, and antioxidant properties make them fit in various industries (Silva et al. 2003). Figure 1 illustrates multiple EO’s plant sources with antifungal properties.

EOs offer a natural, non-surgical treatment for fungal infections, particularly targeting *Aspergillus niger, Candida albicans,* and dermatophyte species (D'Auria et al. 2005; Jahani et al. 2020). Compounds like thymol and carvacrol exhibit potent antifungal activity. Dilution with carrier oils enhances their absorption, stability, and bioavailability (Johnson et al. 2004). EOs are generally considered cost-effective, safe, and have minimal side effects when used topically. They are widely applied for targeted delivery in antibacterial and anti-inflammatory applications to alleviate infection symptoms (Silva et al. 2003). Additionally, EOs exhibit synergistic effects with other natural compounds, such as tea tree and oregano oils, further increasing their antimicrobial efficacy and offering a convenient treatment option, especially for people who rely on natural remedies (Cavanagh And Wilkinson 2002). In summary, due to their historical significance, EOs offer diverse applications and safety profiles, making them a potential alternative to conventional therapies for treating fungal infections and other ailments.

## Chemical nature of essential oil and its mechanism of action

In summary, due to their historical significance, EOs offer diverse applications and safety profiles, making them a potential alternative to conventional therapies for treating fungal infections and other ailments (Pensuk et al. 2007; Zhang et al. 2012; Sadgrove And Jones 2015). More recent and efficient approaches involve supercritical fluid extraction, solvent-free extraction, solar energy-assisted hydrodistillation, and ultrasound-assisted hydrodistillation (Fornari et al. 2012; Bayramoglu et al. 2008; Soni And Kalbande 2024; Zorga et al. 2020). In general, EO extraction consists of the following steps: (a) crushing the plant part; (b) extracting the material with a suitable solvent, or by compound entrapment or distillation; (c) removing unwanted components from extracts; and (d) conducting a suitable method of product analysis (Asl And Khajenoori 2021).

Chemical characterization of EOs involves analytical techniques for qualitative and quantitative analysis of their components. The most commonly used method is Gas chromatography (GC), often combined with Mass Spectrometry (MS), known as GC–MS. It provides detailed chemical profiling through mass spectral identification and retention-time analysis, separating volatile compounds (Abd El-Kareem et al. 2025). High-Performance Liquid Chromatography (HPLC) is used for non-volatile and thermally sensitive compounds (Puthenvitil And Perumal 2025). To determine molecular structures and detect functional groups, Fourier Transform Infrared Spectroscopy (FTIR) can be used (Pasieczna-Patkowska et al. 2025). Advanced methodologies such as enantioselective gas chromatography and Nuclear Magnetic Resonance (NMR) isolate and analyse chiral compounds and stereoisomers (Smelcerovic et al. 2013).

The chemical composition of EOs determines their antimicrobial properties, fragrance profile, and physicochemical characteristics (Lahlou 2004). The major group of EOs' constituents is terpenes, derived from the mevalonate and methylerythritol phosphate biosynthetic pathways, such as humulene, beta-caryophyllene, pinene, and limonene. Terpenes are further divided into subtypes such as monoterpenes and sesquiterpenes (Fokou et al. 2020; Sadgrove et al. 2022). Another important class is phenylpropanoids, including thymol and eugenol, aromatic chemicals derived from the shikimate biosynthetic pathway (Vogt 2010). Additionally, the shikimate pathway also produces sulfur-containing compounds known as isothiocyanates, such as allyl isothiocyanate and benzyl isothiocyanate (Romeo et al. 2018).

EOs possess notable antifungal activity, mainly due to bioactive compounds such as terpenes and phenylpropanoids (Abd Rashed et al. 2021). Oils from (*Syzygium aromaticum*), peppermint (*Mentha piperita*), tea tree (*Melaleuca alternifolia*), and thyme (*Thymus* sp.) have been extensively studied, especially against *Candida, Aspergillus*, and dermatophytes clinical and ATCC strains (Rajkowska et al. 2017; Witkowska et al. 2016; D’agostino et al. 2019; Abdi-Moghadam et al. 2023).

Monoterpene components such as a-pinene, terpinen-4-ol, 1,8-cineole, and gamma-terpinene possess strong antifungal properties (Vassiliou et al. 2023). These chemicals substantially inhibit various fungus species, making them useful in antifungal treatments and applications (Moumni et al. 2021). Monoterpene components such as a-pinene, terpinen-4-ol, 1,8-cineole, and gamma-terpinene possess strong antifungal properties (Ebadollahi et al. 2020), comprising genera *Thymus, Lavandula*, and *Origanum*, which contain essential antifungal molecules, such as thymol and carvacrol. They are especially powerful against *C. albicans* and *A. fumigatus*, two key concerns for immunocompromised people, and against phytopathogens such as *Fusarium* spp., which cause wilts and blights, and *A. niger*, a food crop contaminant. While their antifungal properties are well studied for human health, they also offer enormous potential in treating plant diseases (Karpiński 2020).

**Fig. 2:**
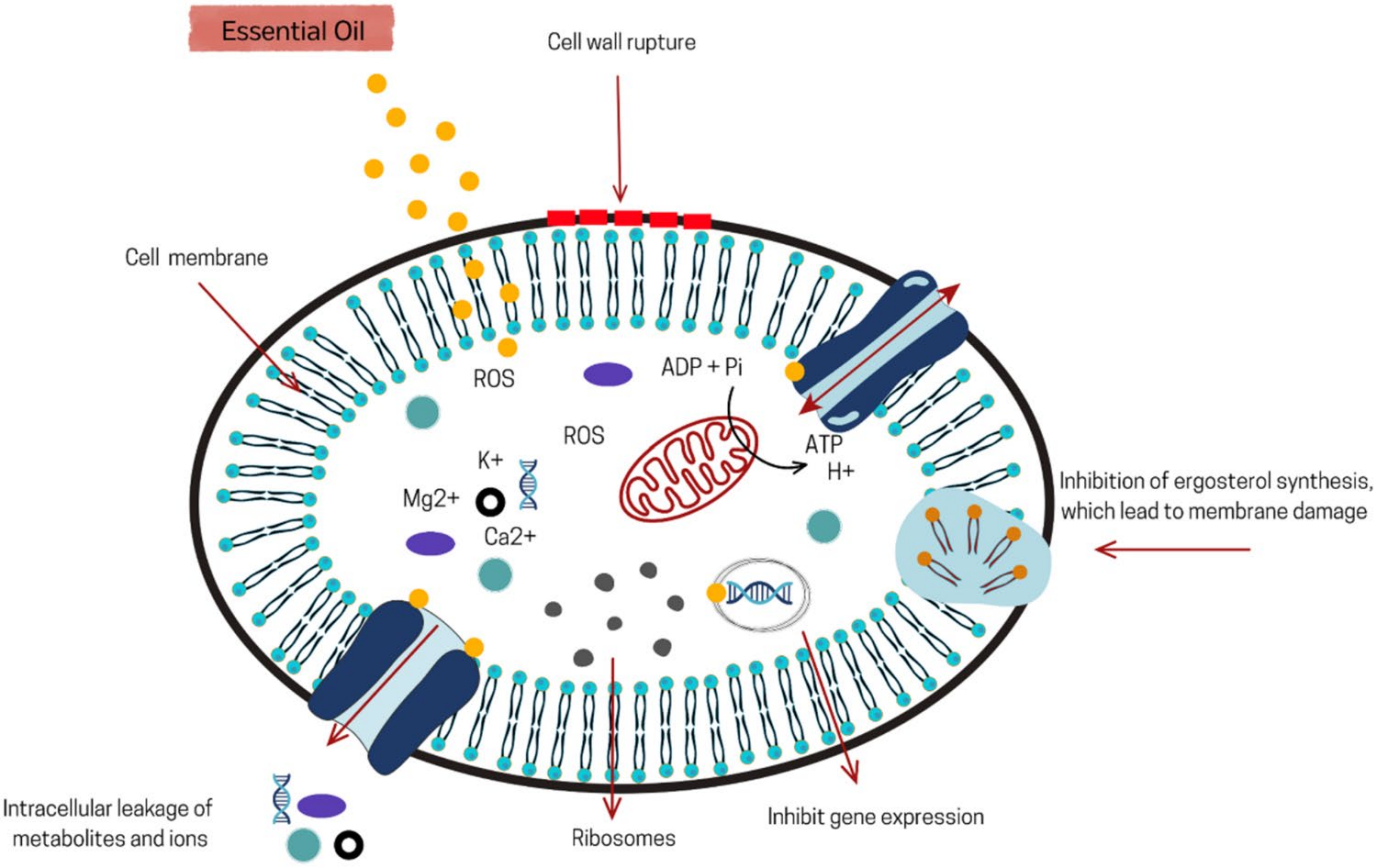
Possible mechanism of action of essential oils on fungal cells

Although the mechanisms of EO antifungal action are not entirely defined, several well-recognized effects include loss of membrane integrity, reduction of ergosterol level, inhibition of cell wall formation, inhibition of gene expression, and suppression of membrane ATPase, as shown in Fig. 2 (Abd Rashed et al. 2021). For example, thymol and carvacrol exhibit antifungal activity by inhibiting ergosterol synthesis and disrupting the membrane structure of *Candida* and *Aspergillus* (Castro et al. 2015; Ahmad et al. 2014). Table 3 summarizes the target fungus species, the active components of different EOs, the chemical classes, and the modes of action of these biomolecules that have already been investigated.

**Table 3:** Details of different EOs, including their major active compound, chemical class, Mechanism of action, and Target fungal species

Oil	Scientific name	Major active ingredient	Chemical class	MOA	Target Fungal Species
Tea Tree	*Melaleuca alternifolia*		Monoterpene	Causes fungal hyphae to degenerate; ergosterol biosynthesis (Yu et al. 2015)	*Botrytis cinerea* (Vassiliou et al. 2023; Yu et al. 2015) *A. ochraceus, A. niger, A. fumiagtus* (Kong et al. 2019; An et al. 2019; Allizond et al. 2023) *C.* albicans (Hammer et al. 1998) *C. auris* (Nenoff et al. 1996) *T. rubrum, T. mentagrophytes* (Carmo et al. 2021; Marcos-Tejedor et al. 2021) *P. chrysogenum*, *P. expansum* (Li et al. 2017; Rocha Neto et al. 2019) *Stachybotrys chartarum* (Pečiulytė 2005) *Malassezia furfur* (Hammer et al. 2000)
Lavender	*Lavandula angustifolia*		Monoterpene	Interacts with ergosterol (Medeiros et al. 2022)	*Aspergillus* sp. (Allizond et al. 2023) *F. solani* (Rashad et al. 2021) *C. albicans* (D'Auria et al. 2005) *Penicillium* sp. (Wińska et al. 2019) *Aureobasidium pullulans* (Wińska et al. 2019)
Clove	*Syzygium aromaticum*		Phenylpropanoids	Membrane binding and permeability alteration (Sharma et al. 2024; Nogueira Sobrinho et al. 2020)	*A. niger, A*. *flavus* *F. oxysporum* (Muñoz Castellanos, et al. 2020) *Botrytis cinérea* (Wang et al. 2010) *Penicillium* sp. (Martínez and González 2013) *Candida* sp. (Biernasiuk et al. 2022) *Rhizoctonia solani* (Wang et al. 2021) *Trichothecium* sp. (Yingprasert et al. 2015) *Aphanomyces* *euteiches, Botrytis cinerea, Colletotrichum lentis, Didymella* *pisi, D. rabiei, D* *lentis, Fusarium* *avenaceum, Stemphylium beticola, Sclerotinia sclerotiorum, Pythium sylvaticum* (Parikh et al. 2021)
			Sesquiterpene		
Oregano	*Origanum vulgare L*		Monoterpene	Ergosterol biosynthesis (Castro et al. 2015; Niu et al. 2020)	*A. flavus, A. terréus* (Kocić-Tanackov, et al. 2012) *P. expansum* (Yingprasert et al. 2015) *Cladosporium* sp*.* (Kapustová et al. 2021) *F. graminearum, Fusarium oxysporum* (Karapetsi et al. 2022) *C. albicans, C. glabrata, and C. riferi* (Niu et al. 2020; Karpiński et al. 2023; Hacioglu et al. 2021) *E. herbariorum, E. rubrum* (Kocić-Tanackov, et al. 2011) *Aphanomyces* *euteiches, Botrytis* *cinerea, Colletotrichum lentis, Didymella pisi, D. rabii, D. lentis, Fusarium avenaceum, Stemphylium beticola, Sclerotinia* *sclerotiorum, Pythium sylvaticum* (Parikh et al. 2021)
Eucalyptus	*Eucalyptus globulus*		Monoterpene	Disrupts fungal cell membranes (Baptista et al. 2015)	*C. albicans* (Bokaeian et al. 2010; Elaissi et al. 2012) *Scopulariopsis brevicaulis* (Elaissi et al. 2012) *Saccharomyces cerevisiae* (Ben Marzoug et al. 2010) *A. niger, A. clavatus* (Su et al. 2006) *Sporobolomyces* sp. (Alitonou et al. 2004) *Rhizopus solani* (Ghaffar et al. 2015) *M. canis* (Elaissi et al. 2012) *Deightoniella torulosa* (Camara, et al. 2010) *F. oxysporum* (Sharma, et al. 1322)
Thyme	*Thymus vulgaris*		Monoterpene	Ergosterol synthesis; thymol disrupts fungal cell membranes (Qi, et al. 2023)	*C. albicans* (Karpiński et al. 2023; Alshaikh And Perveen 2021; de Almeida 2012) *C. auris* (Fernandes et al. 2022) *Cryptococcus neoformans* (Nunes et al. 2016) *A. flavus, A. fumigatus, A. niger* (Witkowska et al. 2016) *Fusarium* sp. (Martins And Bicas 2024) *P. italicum, P. digitatum* (Pinto, et al. 2020) *Alternaria linariae* (Saltos-Rezabala et al. 2022) *Rhizopus stolonifera* (Yan et al. 2021) *Aphanomyces* *euteiches, Botrytis* *cinerea, Colletotrichumlentis, Didymella* *pisi, D. rabiei, D* *lentis, F* *avenaceum, Stemphylium beticola, Sclerotia sclerotiorum, Pymsylvaticum* (Parikh et al. 2021)
Manuka	*Leptospermum scoparium*		Triketone	Inhibits the enzyme 4-hydroxyphenylpyruvate dioxygenase (HPPD) (Mallet et al. 2019)	*Malassezia furfur* *Trichosporon mucoides* *C. albicans* *C. tropicalis* *C. glabrata,* *Phytophthora cactorum* *Cryphonectria parasitica* (Mathew et al. 2020)
			Sesquiterpene	Not found	
			Monoterpene	Not found	
Peppermint	*Mentha piperita*		Monoterpene	Membrane integrity, inhibiting hyphal growth by inducing oxidative stress, cell cycle arrest, and apoptosis (Zore et al. 2022)	*A. flavus* (Abd El-Hack et al. 2023) *Fusarium sambucinum* (Pérez‐Vázquez et al. 2022) *Candida albicans* (Rajkowska et al. 2017; Karpiński et al. 2023; Zore et al. 2022)
				Membrane integrity (Samber et al. 2015)	
Lemongrass	*Cymbopogon citratus*		Monoterpene	Membrane integrity and synthesis; inhibit spore germination, proliferation, and cellular respiration (Leite et al. 2014)	*C. albicans, C. glabrata, C.krusei, C.parapsilosis, C. tropicalis, C. oleophila* (Leite et al. 2014; Leite et al. 2015; Silva et al. 2008; Sharma et al. 2016; Singh et al. 2016; Powers et al. 2018; Irkin And Korukluoglu 2009) *C. neoformans* (Powers et al. 2018) *A. fumigatus, A. niger* (Powers et al. 2018; Irkin And Korukluoglu 2009) *Colletotrichum gloeosporioides* (Lozada, et al. 2019) *Penicillium* sp. (Premathilake et al. 2018) *Eurotium repens* (Correa-Pacheco et al. 2018) *F. oxysporum, Alternaria alternata, Hansenula anomala* (Irkin And Korukluoglu 2009; Premathilake et al. 2018; Debonne et al. 2022; Gangavarapu And Palwai 2021) *P. roquefortii,* *S. cerevisiae, S. uvarum, Schizosaccharomyces pombe,* *Metschnikowia* *fructicola* (Irkin And Korukluoglu 2009) *Aphanomyces* *euteiches, Botrytis* *cinerea, Colletotrichumlentis, Didea* *pisi, D. rabiei, D* *lentis, Fusarium* *avenaceum, Stemphylium beticola, Sclerotia* *sclerotiorum, Pythium sylvaticum* (Parikh et al. 2021)
			Monoterpene	Ergosterol biosynthesis; inhibition of plasma membrane ATPase (PM-ATPase) enzyme (Sharma et al. 2016)	
			Monoterpene	Ergosterol metabolism (OuYang et al. 2021)	
			Sesquiterpene	Permeabilization and membrane integrity (Noge And Becerra 2009)	
			Monoterpene	Geranyl acetate can undergo photooxygenation, resulting in the formation of various epoxides and hydroperoxides, which have been shown to exhibit antifungal activity (Khayyat And Sameeh 2018)	
Neem	*Azadirachta indica*		Limonoids	Ergosterol biosynthesis (Ospina Salazar et al. 2015)	*R. solani* (Ali et al. 2017) *Sclerotium rolfsii, Sclerotinia sclerotiorum* (Council And Neem 2002) *F. oxysporum* (Murugesan 2024)
		Nimbolinin, nimbin, nimbidin, nimbidol, gedunin, salannin	Limonoids and triperpenoids	Not fully understood	
			Flavonoid	Disruption of the plasma membrane and inhibition of nucleic acid synthesis, protein synthesis, and mitochondrial functions (Nguyen And Bhattacharya 2022)	
Geranium	*Pelargonium graveolens*		Monoterpene	Ergosterol biosynthesis (Pereira Fde et al. 2015)	*F. proliferatum* (Grine, et al. 2023) *F. solani* *Macrophomina phaseolina* *Rhizoctonia solani* *Sclerotium rolfsii* (Abdel-Kader et al. 2011) *C. albicans* (Medeiros et al. 2022; Zore et al. 2011) *Pythium ultimum* (Desilets et al. 1994) *A. flavus, A. ochraceus* (Tang et al. 2018)
Lemon Balm	*Melissa officinalis*		Monoterpene	Ergosterol biosynthesis; inhibits plasma membrane ATPase (PM-ATPase) (Sharma et al. 2016)	*F. oxysporum albedinis, F. oxysporum lini* *Mucor ramannianus* *C. albicans* (Karpiński et al. 2023; Abdellatif et al. 2014)
			Monoterpene	Sterol biosynthesis (Ghannoum And Rice 1999)	
			Monoterpene	Disrupts membrane homeostasis (Singh et al. 2016)	
Lime	*Citrus aurantifolia*		Monoterpene	Cell wall and cell membrane damage; DNA damage; apoptosis (Leite-Andrade, et al. 2022)	*A. flavus* (Hassanein, et al. 2023) *C.* albicans (Fekrazad, et al. 2015)
			Monoterpene	Cell wall damage (Macêdo Andrade et al. 2018)	
			Monoterpene	Decreases mycotoxin production; fungal cell membrane disruption; block membrane synthesis; inhibition of the spore germination, fungal proliferation, and cellular respiration (Qi, et al. 2023)	
Myrrh Oil	*Commiphora myrrha*		Sesquiterpene	Not Described	*C. tropicalis, C. albicans, C. laurentii* (Nikolic et al. 2016; Omer et al. 2011; Alabdalall 2023) *Acremonium strictum* *A. flavus, A. sydowii* *Chaetomium globosum,* *C. cladosporioides,* *C. sphaerospermium* *Cladosporium* sp.*,* *Cochliobolus spicifer,* *Drechslera biseptata,* *Embellisia chlamydospore, Eurotium amstlodami,* *Fusarium semitectium,* *Myceliophthora lutea,* *P. chrysogenum, P. fellutanum, P. reticulosum,* *Phoma tropica,* *Torula caligans,* *Trichoderma psudokoningii,* *Ulocladium consortia* (Al-Sabri, et al. 2014)
				Membrane disruption and ergosterol biosynthesis affect succinate dehydrogenase (SDH) and NADH oxidase enzymes (Chen et al. 2018)	
Niaouli	*Melaleuca quinquinervia*		Monoterpene	Aflatoxin biosynthetic pathway (Kim et al. 2018), biofilm and cell membrane (Pries et al. 2023)	*C. auris* (Fernandes et al. 2022) *A. flavus* (Kim et al. 2018)
			Sesquiterpene	Not found	
			Monoterpene	Interacts with ergosterol (Barros, et al. 2023)	
Palmarosa	*Cymbopogon martinii*		Monoterpene	Leakage of cell wall, ROS production (Tang et al. 2018), ergosterol (Leite et al. 2015)	*A. niger, P. chrysogenum, F. acuminatum, P. chrysosporium* (Abdi-Moghadam et al. 2023*), C. albicans* (de Almeida 2012) *Botrytis cinerea* (Kou et al. 2023) *Phomopsis sp., F. solani, S. rolfsii, Macrophomina phaseolina Aphanomyces euteiches Colletotrichum lentis, Didymella pisi, D. rabiei, D. lentis, F. avenaceum, Stemphylium beticola, S. sclerotiorum, Pythium sylvaticum* (Parikh et al. 2021; Xavier et al. 2020) *P. expansum* (Rocha Neto et al. 2019)
			Monoterpene	Damages in the plasma membrane and cell wall (Medeiros et al. 2022)	
Patchouli	*Pogostemon cablin* Benth		Sesquiterpene	Cell wall and plasma membrane damage (Cunha et al. 2023)	*C. albicans* (Cunha et al. 2023)
Rosemary	*Rosmarinus officinalis*		Monoterpene	Inhibit aflatoxin biosynthetic pathway (Kim et al. 2018), penetrate biofilm (Pries et al. 2023), cell membrane permeabilization (Hoch et al. 2023)	*A. flavus* (Kim et al. 2018; Moghtader et al. 2011; Mojtaba et al. 2016) *C. albicans* (Karpiński et al. 2023; Mojtaba et al. 2016; Murtiastutik, et al. 2023) *E. floccosum* *T. verrucosum* (Mojtaba et al. 2016) *Geotrichum candidum* *Sclerotinia nivalis Cylindrocarpon destructans* *Alternaria panax* *Botrytis cinerea* *Fusarium oxysporum* (Hussein et al. 2020; Özcan And Chalchat 2008) *Fusarium* sp*.* (Özcan And Chalchat 2008)
			Monoterpene	Interact with ergosterol (Barros, et al. 2023)	
			Monoterpene	Biosynthesis of cell wall and plasma membrane (Medeiros et al. 2022)	
			Monoterpene	Membrane disruption (Kong et al. 2022)	
			Monoterpene	Affect and damage the cell wall structure (Yamaguchi et al. 2009)	
			Monoterpene	Not fully understood (Leite-Andrade, et al. 2022), probably cell wall and membrane damage (Thakre et al. 2018)	*Alternaria alternate* (Özcan And Chalchat 2008)
Sandalwood	*Santalum album* L		Sesquiterpene	Disrupt microtubule (Kim et al. 2017)	*C. neoformans,* *A. niger, C. albicans* (Powers et al. 2018) *Madurella mycetomatis* (Abd Algaffar et al. 2024) *M. canis* (Nardoni et al. 2015)
				Ergosterol biosynthesis (Sousa et al. 2023b)	*M. mycetomatis* (Abd Algaffar et al. 2024)
Tangerine	*Citrus reticulata*		Monoterpene	Membrane permeability; ion homeostasis, anabolism, and catabolism disruption (Yu et al. 2022)	*C. parapsilosis* (Leite-Andrade, et al. 2022), *C. tropicalis* (Yu et al. 2022), *A. carbonarius, A. ochraceous, A. fumigatus, A. parasiticus, A. niger,* and *A. nomius* (Sabry et al. 2024) *P. chrysogenum* (Matan And Matan 2008)
			Monoterpene	Cell wall disruption; ROS accumulation; inhibition of key enzymes (Nazzaro et al. 2017)	
			Monoterpene	Disrupts cell membrane and cell wall biosynthesis (Nazzaro et al. 2017)	
			Alkylbenzene	Not found	
Turmeric	*Curcuma longa* L		Sesquiterpene	Structural modifications in fungal cells; functional changes (Orellana-Paucar And Machado-Orellana 2022)	*C. tropicalis* (Orellana-Paucar And Machado-Orellana 2022) *P. notatum* *A. fumigatus, A. niger, A. flavus* *T. rubrum, T. violaceum, T. mentagrophytes* *E. floccosum* *M. gypseum,* *Sporothrix schenckii* (Orellana-Paucar And Machado-Orellana 2022) *R. solani,* *Phytophthora infestans* *Erysiphe graminis* *Puccinia recondite, B. cinerea,* *F. solani,* *Helminthosporium oryzae* (Zorofchian Moghadamtousi et al. 2014) *F. graminearum* (Chen et al. 2018)
Vetiver	*Vetiveria zizanioides*		Sesquiterpene	Not found	*C. albicans, C. glabrata* (Burger et al. 2017) *A. niger, C. neoformans* (Powers et al. 2018)
Yarrow	*Achillea millefolium*		Monoterpene	Disrupts cell membrane; inhibits ATPases; alters cell wall biosynthesis (Nazzaro et al. 2017)	*A. niger, P. hirsutum* (Fierascu et al. 2015) *C. albicans, C. tropicalis, C. parapsilosis, S. cerevisiae* (Aydin And Sevindik 2018)
			Monoterpene	Aflatoxin biosynthetic pathway (Kim et al. 2018), penetrates biofilm (Pries et al. 2023), and cell membrane permeabilization (Hoch et al. 2023)	*C. auris* (Fernandes et al. 2022)
			Sesquiterpene	Disrupt cell membrane (Nogueira Sobrinho et al. 2020)	*S. sclerotiorum*, *F. oxysporum* *A. brassicicola* (Hilgers et al. 2021)
			Monoterpene	Decreases metabolic activity, inhibits biofilm (Nogueira Sobrinho et al. 2020)	

## Essential oil-based nanoformulations for the management of dermatophytosis: types, mechanisms, and therapeutic potential

Nanotechnology-based drug delivery systems (NDSs) are based on encapsulating active compounds in nanoparticles (10–1000 nm) to improve drug targeting and delivery for disease treatment and detection (Roostaee And Sheikhshoaie 2022). These systems increase efficacy, stability, and bioavailability, while reducing side effects (Sharma et al. 2019). Nanoparticles smaller than 100 nm demonstrate enhanced tissue penetration and accumulation at the target sites, a phenomenon known as the enhanced permeation and retention (EPR) effect in diseased tissues (Barua And Mitragotri 2014; Ngoune et al. 2016; Ejigah et al. 2022). Surface modification further improves targeted drug delivery by extending bioavailability and minimizing immune detection of nanoparticles, such as through PEGylation (Nolte et al. 2023).

A variety of methodologies are used to synthesize nanoformulations, generally classified into top-down and bottom-up approaches. In top-down synthesis, bulk materials are reduced to nanoscale sizes and shapes by advanced techniques such as milling, lithography, or laser ablation. In contrast, bottom-up approaches use chemical, physical, or biological processes to make nanoparticles from atomic or molecular building blocks. These processes often include sol–gel reactions, microemulsion, and hydrothermal synthesis (Chattha et al. 2023). Recently, green synthesis using plant extract or microorganisms has gained attention due to their sustainability and environmentally friendly approach. These techniques utilize biological agents within cells to facilitate nanoparticle formation, eliminating the need for external capping agents or toxic chemicals (Ying et al. 2022). Overall, the selected synthesis method and stabilizing agent play an essential role in defining the properties and performance of the nanoformulation.

**Fig. 3:**
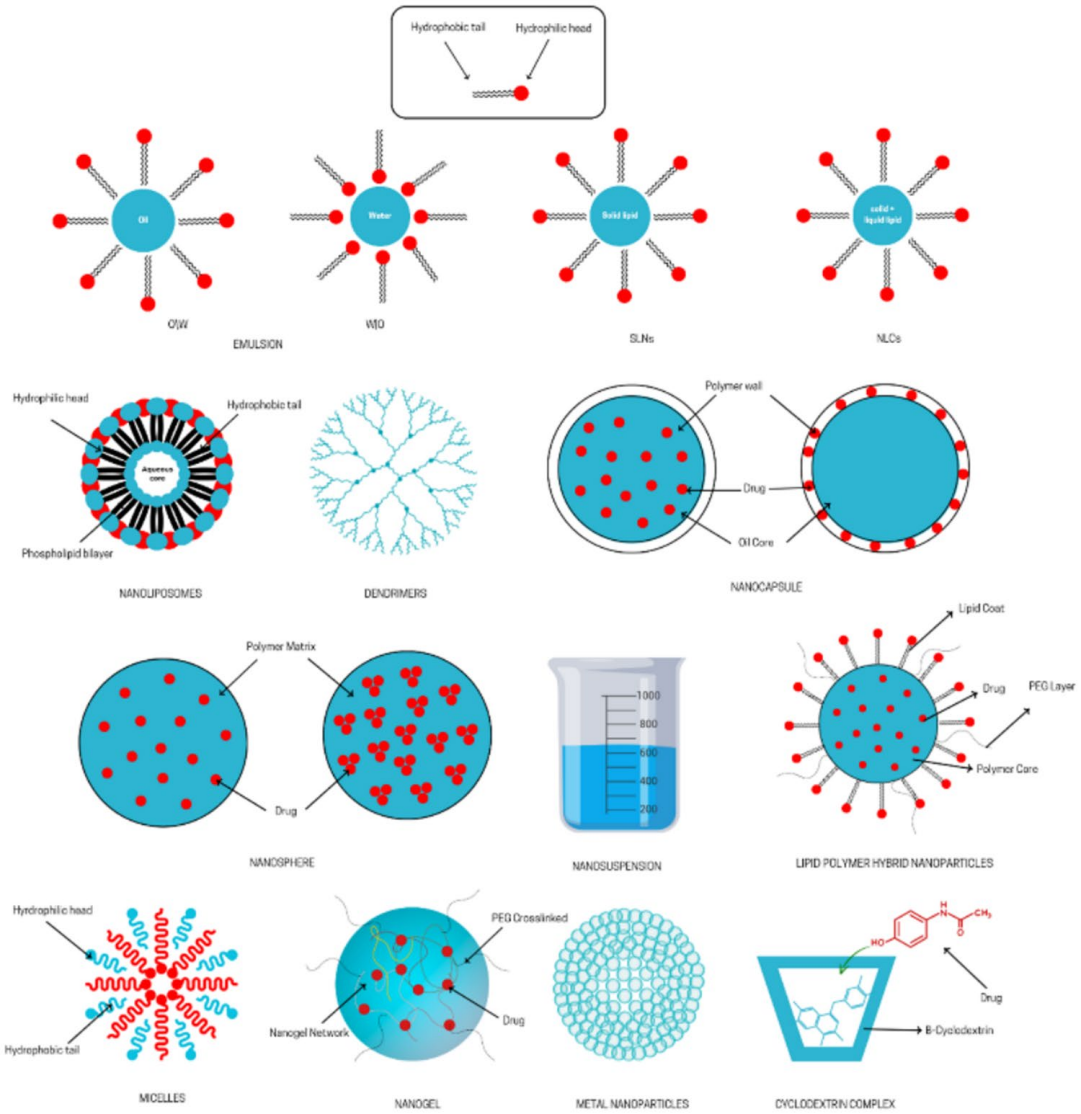
Types of nanoformulations containing essential oils with described antifungal activity. Red solid spheres: drug/essential oil

Different types of NDSs have been studied for their potential to treat dermatophyte infections, including liposomes, niosomes, nanoemulsions, solid lipid nanoparticles, dendrimers, nanospheres, and other lipid-based nanocarriers (Stefanov And Andonova 2021; Tarannum et al. 2024). Figure 3 illustrates different types of nanoformulation. These NDSs may improve the topical delivery of EOs, potentially enhancing their effectiveness against dermatophytes; however, most have been used primarily in combination with conventional antifungal drugs. Nanoformulations of fluconazole and terbinafine have demonstrated significantly lower minimum inhibitory concentrations (MICs) against dermatophytes than their traditional counterparts, indicating enhanced antifungal efficacy and improved therapeutic outcomes (Bafrui et al. 2020).

For alternative treatments, nanoformulations significantly enhance EOs' properties by increasing surface area, water solubility, and skin penetration, while also providing controlled release and protection against degradation (Bafrui et al. 2020; Sousa et al. 2023a). Encapsulation of EOs enhances their stability and solubility through various formulation methods, leveraging EOs' antimicrobial, anti-inflammatory, and antioxidant benefits for different applications (São Pedro et al. 2013). Despite these advantages, specific research on the use of EO-based NDSs for dermatophyte infections is limited, and further studies are needed to evaluate their therapeutic effect.

### Nanoemulsion

Emulsions, such as water-in-oil (W/O) and oil-in-water (O/W), are commonly used, while more complex systems, such as W/O/W and O/W/O, have been developed to encapsulate bioactive substances (David et al. 2023). Across studies, a reduction in droplet size consistently enhanced the physicochemical properties and bioactivity of emulsions. EO-containing nanoemulsions offer benefits such as protection against oxidation, increased solubility, controlled release, and enhanced antimicrobial efficacy.

Several studies have reported the successful formulation of plant extract-based nanoemulsions for the treatment of dermatophytic infections, with nanoemulsions generally outperforming their corresponding bulk extract or oils. For instance, a sodium alginate-stabilized nanoemulsion of *Phyllanthus niruri* methanolic extract showed potent inhibition against *T. rubrum* (18.5 ± 0.4 mm zone) and *T. mentagrophytes* (18.0 ± 0.7 mm zone) at 100 μL/well, compared with the extract alone (13.3 ± 0.5 mm and 12.1 ± 0.7 mm, respectively) (Pathania et al. 2022). The enhancement was attributed to the small droplet size, which facilitated penetration through fungal lipid barriers and increased fungal susceptibility. At the same time, bioactive constituents in *P. niruri*, such as flavonoids, triterpenoids, phyllanthin, glycosides, and tannins, were responsible for the antimicrobial effects. Notably, the nanoformulation enabled sustained drug release (93% over 12 h, following first-order kinetics) and enhanced bioavailability (Paim et al. 2018).

Kumar et al. evaluated a blended cinnamon oil and usnic acid nanoemulsion (CUN) against *T. mentagrophytes,* demonstrating both in vitro and in vivo models. In vitro cup-plate method showed a maximum zone of inhibition of 1.19 cm at 10 mg/mL after 48 h; the MIC and MFC values were 55 μg/mL and 60 μg/mL, respectively. Notably, in a guinea pig model of trichophytosis, topical application of the CUN gel yielded superior clinical efficacy and mycological cure rate compared with 8% ciclopirox. This enhanced efficacy was attributed to improved skin penetration, increased surface area for interaction with fungal cells, and a synergistic mechanism involving cytoplasmic membrane disruption and enzymatic inactivation (Ramteke et al. 2019).

Similarly, Moazeni et al. investigated that *T. vulgaris* EO nanoemulsion (TV EO-NE) significantly improved antifungal potency against multiple dermatophyte species, reducing the MIC to 0.016 μg/mL compared with the bulk oil (0.031–0.0625 μg/mL). This activity was primarily driven by thymol (22.10%) and carvacrol (13.02%), which exhibited improved membrane penetration in the nanoform and induced fungal plasma membrane disruption and apoptosis, a form of programmed cell death (Moazeni et al. 2021). Comparable trends were observed in Danielli et al., where *Stenachaenium megapotamicum* volatile oil nanoemulsion against *E. floccosum and T. rubrum*. The pure oil showed MIC values of 20.81–166.25 μg/mL and an MFC of 166.25 μg/mL, while the nanoemulsion significantly reduced MICs to 5.18–41.5 μg/mL and MFCs to 5.18–41.5 μg/mL. The nanoemulsion enhanced solubility, stability, and passive transport, while hydrophobic constituents, predominantly thymol (10.8%), accumulated within fungal membranes, causing membrane rupture, increased permeability, and ergosterol disruption (Danielli et al. 2013).

Kotwiski et al. formulated a nanoemulsion containing *Lippia origanosides* EO and tested it against *M. canis, T. tonsurans, T. mentagrophytes,* and *E. floccosum*. They observed MICs ranging from 11.2 to 234.4 µg/mL. The antifungal mechanism remained consistent, with carvacrol and thymol disrupting fungal cytoplasmic membrane integrity by increasing permeability and inhibiting ergosterol biosynthesis, leading to leakage of intracellular components and cell death. The nanoemulsion system enhances this effect by improving EO solubility, stability, and contact with the fungal membrane (Kotwiski et al. 2024). The most noticeable antifungal activity was observed in combination nanoemulsions as demonstrated by Al-Suwaytee et al., where an oil-in-water nanoemulsion formulation combines voriconazole (VCZ) with *Pinus sylvestris* L. EO (PSEO). This NE demonstrated superior antifungal activity against the *M. canis*, yielding an inhibition zone diameter of 80.33 ± 4.61 mm in disk agar diffusion assays (5 μL applied), outperforming VCZ suspension alone (61.66 ± 1.52 mm) and PSEO alone (48.33 ± 1.52 mm), with no activity from blank NE. The enhanced efficacy resulting from synergistic mechanisms, including VCZ-mediated inhibition of ergosterol biosynthesis, EO-induced membrane destabilization, impaired mitochondrial and DNA function, efflux pump inhibition, and improved drug penetration, is amplified by the nanoemulsion’s nanoscale droplets (Al-Suwaytee et al. 2024).

### Nanosphere

A nanosphere is a uniform, dense matrix that can transport active compounds within or on its surface. It can be developed from different materials, such as polymers, carbon, silica, and metal–organic frameworks (MOFs) (Muttaqien et al. 2022). Nanospheres enable sustained and controlled EO release (Abd Rashed et al. 2021; Abdi-Moghadam et al. 2023), site-specific delivery, minimize side effects, and improve their therapeutic efficacy (Muttaqien et al. 2022). The biocompatibility and biodegradability of nanosphere materials contribute to safety and better tolerance.

Carmo et al. compared the antifungal activity of free ketoconazole (KTZ; MIC 0.03–2.00 mg/mL) with that of EO-loaded gold nanospheres formulated with Fragonia EO (AuNPFRA) and tea tree oil (AuNPTTO). The EO-loaded gold nanospheres showed improved antifungal potency against *T. interdigitale* with MIC values of 0.06–0.25 mg/mL for Fragonia oil and 0.12–0.50 mg/mL for tea tree oil. These nanosphere formulations enhanced EO delivery and showed fungistatic activity by disrupting fungal cell membrane fluidity and permeability via terpene-mediated mechanisms. Notably, treatment with AuNPFRA and AuNPTTO resulted in increased antifungal inhibition and reduced inflammatory responses compared with the corresponding free essential oils (Carmo et al. 2021).

### Polymeric nanocapsule

A polymeric nanocapule consists of a liquid or solid core coated with a polymeric shell, typically made from natural or synthetic materials such as poly-e-caprolactone (PCL), poly (lactide) (PLA), or poly (Lactide-co-glicolide) (PLGA) (Couvreur et al. 2002).

An in vitro study conducted by Flores et al. compared TTO-loaded nanocapsules with nanoemulsions and emulsions using a nail infection model against *T. rubrum.* While emulsions were the least effective and nanoemulsions showed only moderate efficacy, nanocapsules achieved a significant reduction in fungal load to 1.0 log CFU/mL within 7 days. They maintained minimal regrowth at 14 days, highlighting their surprising sustained release and enhanced tissue penetration (Flores et al. 2013).

Dos Santos et al. developed a dual-loaded nanocapsule against dermatophytes, including *T. rubrum*, *T. mentagrophytes,* and *M. canis,* by incorporating terbinafine hydrochloride (TBF) and ozonated sunflower seed oil (OZ). Mechanistically, terbinafine (TBF) inhibits squalene epoxidase in the ergosterol biosynthesis pathway, leading to disruption of fungal cell membrane integrity. In parallel, ozone (OZ) generates reactive oxygen species through ozonide formation, which oxidize key microbial cellular components, including double bonds and sulfhydryl groups. The combined actions of TBF and OZ enhance antifungal efficacy through complementary mechanisms. In vitro, this synergy resulted in minimum inhibitory concentrations (MICs) that were 20–200 times lower than those of TBF solution alone (0.0011–0.0381 μg/mL vs. 0.0625–0.250 μg/mL). Moreover, the formulation exhibited fungicidal activity, achieving a 99.9% reduction in fungal viability within 96 h at a TBF-equivalent concentration of 0.0011 μg/mL (Dos Santos et al. 2023).

### Nanoliposomes

Nanoliposomes, composed of phospholipids such as lecithin, can deliver both hydrophobic and hydrophilic drugs by improving solubility, stability, and site-specific delivery. Their use in dermatophyte treatment has enhanced efficacy while reducing adverse effects (Mousavi et al. 2023; Garg et al. 2020). In a study by Aguilar-Pérez et al., prepared oregano EO (OEO) nanoliposomes were prepared via thin-film hydration-sonication. These nanoliposomes exhibited superior antifungal activity against the *T. rubrum*, achieving 81.66 ± 0.86% mycelial growth inhibition (MGI) at 1.5 μL/mL (tested via agar dilution on potato dextrose agar at 25 °C for 3 days across doses of 0.25, 0.5, 1.0, 1.5 μL/mL, vs. free OEO at 40.1 ± 2.16%). OEO components, such as carvacrol or thymol, interact hydrophobically with lecithin acyl chains, thereby increasing membrane rigidity and enabling sustained release. Hydrogen bonding between EO hydroxyls and phospholipid phosphate heads enhances entrapment and improves antifungal efficacy compared to free oil (Aguilar-Pérez et al. 2021).

### Niosomes

Niosomes are vesicles formed by non-ionic surfactants, such as Span and Tween, capable of encapsulating hydrophilic and hydrophobic drugs, and are more stable and cost-effective than nanoliposomes (Gorjian et al. 2021). Haghani et al. (2025) formulated a 1% (w/v) green niosomal piperlongumine (PL) gel as a topical treatment for terbinafine-resistant *T. indotineae* in a guinea pig model. Guinea pigs received 1 g of the gel topically twice daily (every 12 h) for 28 days and abraded back inoculated with 10^8^ cells/mL *T. indotineae* spores. The 1% PL niosomal gel achieved complete mycological cure by day 28 and significantly reduced clinical lesion scores, epidermal thickness, and inflammation compared with terbinafine, with lower cytotoxicity than free PL. PL exerts antifungal effects through ROS production, SQLE/CYP51 enzyme inhibition, and NLRP3 inflammasome suppression, enhanced by niosomal skin penetration and sustained release. This nanoformulation offers a promising alternative for the treatment of resistant dermatophyte infections, warranting clinical translation (Haghani et al. 2025).

### Solid lipid nanoparticles (SLNs)

SLNs improve drug penetration and produce sustained effect, and have potential for antifungal drug delivery. Several studies have demonstrated the efficacy of SLNs against dermatophytes and bacteria. Svetlichny et al. developed SLNs loaded with copaiba oil, with and without allantoin, that showed enhanced antifungal activity against *T. rubrum* and *M. canis*, with lower MIC_90_ values (e.g., 1.95–500 μg/mL) compared to free oil, further improved by allantoin. Mechanistically, the antifungal effect results from disruption of fungal membrane integrity, leading to altered membrane permeability and cell death, similar to the action of polyene antifungals (Svetlichny et al. 2015). The study supports the use of essential oil nanoencapsulation as a strategy for treating cutaneous fungal infections.

### Nanostructured lipid carriers (NLCs)

NLCs, mainly composed of both kinds of lipids, solid and liquid, produced a sustained and prolonged effect compared to the SLNs (Li et al. 2020). They provide enhanced stability, reduced toxicity, and improved bioavailability, making them unique for application in gene, cancer, and brain-targeted delivery (Tang et al. 2023; Kanojia et al. 2022). Fard et al. conducted a randomized, double-anonymized, placebo-controlled trial to evaluate the efficacy of *Zataria multiflora*-loaded nanostructured lipid carrier (ZM-NLC) topical gel in treating mild to moderate cutaneous dermatophytosis. The ZM-NLC gel (1% w/w) significantly reduced inflammation (87.5%), itching (90%), scaling (95%), and lesion size after 4 weeks, outperforming placebo and achieving faster mycological clearance. *T. mentagrophytes,* and *T. interdigitale* complex prevailed among the isolates, confirming NLCs' efficacy in enhancing essential oil delivery without causing toxicity (Fard et al. 2023).

### Cyclodextrin complex

Cyclodextrins are cyclic oligosaccharides composed of glucose units linked by α−1,4-glycosidic bonds (Carneiro et al. 2019). They can be used to encapsulate hydrophobic molecules due to their unique structure, improving water solubility and stability for drug delivery applications (Shalaby et al. 2021; Boczar And Michalska 2022). Research has shown that cyclodextrins can increase the antifungal activities of EOs.

β-Cyclodextrin (β-CD) encapsulation of *S. montana* EO significantly reduces the growth of dermatophytes like *E. floccosum*, *Microsporum*, and *Trichophyton* species. The 10:90 oil:β-CD ratio was the most effective against dermatophytes at 100 µg/mL. The controlled release of oil volatiles (primarily carvacrol, p-cymene, γ-terpinene, borneol, and thymol) from the β-cyclodextrin cavity enables prolonged fungal contact and membrane disruption without altering the oil's qualitative composition (Haloci et al. 2014). Another study demonstrated that cyclodextrin has the capacity to increase the antifungal properties of *O. vulgare* and *Rosmarinus officinalis* EOs against dermatophytes and observed complete inhibition of *T. violaceum* and *T. tonsurans* by oregano-β-CD (Torres-Alvarez et al. 2020).

### Nanogel

Nanogels, which are colloid-based hydrogels composed of 3D networks of ionic and non-ionic hydrophilic polymers, enhance EO delivery for antifungal applications. Carvalho et al. evaluated *Syzygium aromaticum* and *Cinnamomum* spp. EOs encapsulated in cinnamic acid-grafted chitosan (CS-CA) nanogels against *M. canis*. They observed 100% inhibition at 400 μg/mL (MIC), demonstrating improved antifungal activity compared to free EOs (Carvalho et al. 2021). Encapsulation improved EO stability and bioavailability at the infection site, but the exact mechanistic pathways were not detailed.

### Metal nanoparticles

Metal nanoparticles (MNPs), composed of pure metals such as gold (Au), silver (Ag), and nickel (Ni), range from single atoms to clusters of 1–100 nm (Saleh 2022). Across various studies, MNPs-based systems demonstrated improved antidermatophytic efficacy compared with free (EOs), and considerable variations were observed in potency, mechanism, and translational approach. MNPs act by generating reactive oxygen species (ROS), disrupting membranes, and interfering with metabolic pathways (Moosavy et al. 2023). In addition, EOs components, such as terpenes and phenolic compounds, increase membrane permeability, facilitating metal-ion penetration and intensifying intracellular stress (Begum et al. 2022).

Scandorieiro et al. reported robust activity using combined formulations of oregano and rosemary essential oils with biogenic AgNPs in antifungal nail lacquers, achieving low MIC ranges (0.05–0.25% v/v for EOs and 2.48–6.23 μg/mL for bioAgNPs) against various dermatophyte species, including *T. mentagrophytes*, *T. rubrum, M. canis,* and *M. gypseum*. The synergistic interaction between phenolic EO constituents (e.g., carvacrol and thymol) and Ag⁺ ion release resulted in membrane destruction, cytoplasmic leakage, and structural damage, as confirmed by Scanning Electron Microscopy analysis, pointing out the benefits of combination systems over single-agent approaches (Scandorieiro et al. 2024).

In contrast, studies employing EO-mediated synthesis of AgNPs alone, such as those using *Acanthospermum australe* EO, demonstrated comparatively higher MIC values (≤256 μg/mL) against *M. canis* and *E. floccosum.* The antifungal mechanism primarily involves the controlled release of silver ions, which disrupt microbial membranes, induce oxidative stress, and interfere with essential enzymes, DNA replication, and the respiratory chain, leading to cell death (Mussin And Giusiano 2024). More mechanistically detailed insights were provided by Abdallah et al., who green-synthesized AS-AgNPs using *Achillea santolina* extract, not only inhibited *T. rubrum *in vitro (MIC 128 μg/mL) but also achieved complete fungal clearance in an in vivo model by day 14 following topical application. This study is distinctive for its comprehensive mechanistic evaluation, which included disruption of cell membrane integrity, inhibition of ergosterol biosynthesis via suppression of the CYP51 enzyme, reduced cell wall integrity through inhibition of β−1,3-D-glucan synthase and chitin synthase, ROS accumulation, and apoptosis induction (Abdallah et al. 2023).

Gold nanoparticle-based systems, although less extensively studied, offered complementary advantages. Carmo et al. showed that gold nanospheres loaded with *Taxandria fragrans* and *Melaleuca alternifolia* EOs achieved fungistatic effects against *T. interdigitale* at MICs comparable to those of silver-based systems (0.06–0.50 mg/mL). The terpene-rich composition of these EOs enhanced fungal cell membrane fluidity and permeability, leading to cell disruption (Carmo et al. 2021).

Comparative evaluation of these studies indicates that AgNPs-based nanoformulations exhibit superior antifungal efficacy against dermatophytes via synergistic, multi-target mechanisms. While gold nanoparticle systems are effective in enhancing EO bioavailability and reducing inflammation, they may require further optimization to exhibit comparable fungicidal activity.

## Potential application of the essential oil-based nanoformulations

The search for non-systemic therapies aims to enhance patient safety and has opened the door to new applications for EOs (Carson et al. 2006). A systematic review stated that the topical application of EOs has moderate evidence for the treatment of tinea pedis using tea tree oil (Freeman, et al. 2019). In vitro studies have investigated the antifungal activity of *Artemisia sieberi* EO against superficial fungal infections caused by *Microsporum* spp. and have observed that its antifungal activity is due to α- and β-thujone (Parrish et al. 2020). Mycodin®, a widely available lotion and spray formulation, utilizes thujone from *A. sieberi* (15 mg) to address minor fungal infections. However, the precise mechanism of action against dermatophyte species remains unclear, underscoring the need for further investigation.

A significant advantage of incorporating EOs into topical formulations is their low molecular weight, which enhances skin permeability and increases their ability to reach pathogenic fungi (Albert And Weis 2004). Nevertheless, the lipophilicity and volatility of EOs pose formulation challenges. One effective strategy for improving the antifungal activity of EOs is their incorporation into nanoformulations, enabling lower EO concentrations while maintaining therapeutic efficacy. Nanoencapsulation increases EO availability at the microbial cell membrane by reducing particle size and increasing surface area (Miastkowska et al. 2020). Kaur et al. (2021) discussed the advantages and limitations of antimicrobial nanocarriers, such as nanohydrogels. These nanocarriers, often derived from natural polymers, offer a sustainable approach; however, comprehensive toxicological studies are crucial. Table 4 provides details on the physicochemical properties and antifungal efficacy of the essential oil-loaded nanoformulation against various dermatophyte species.

**Table 4:** List of essential oil-based nanoformulations, physicochemical properties, and corresponding target dermatophyte fungal species

Essential oil	Nanoformulation type	Particle size	PDI*	Encapsulation efficiency	MIC value or ZOI**	Dermatophyte species	References
*Phyllanthus niruri*	Nanoemulsion	192 nm (initial), stable to 234 nm (30 days)	0.2–0.4	NA	18.5 ± 0.4 mm	*T. rubrum*	Paim et al. (2018)
				NA	18.0 ± 0.7 mm	*T. mentagrophytes*	
*Cinnamomum* genera	Nanoemulsion	NA	NA	NA	MIC: 55 μg/mL ZOI: 1.19 cm (10 mg/mL, 48 h)	*T. mentagrophytes*	Ramteke et al. (2019)
*Thymus vulgaris*	Nanoemulsion	127.6 ± 62.52	0.23	NA	0.016–0.0625 μg/mL	*T. tonsurans, T. mentagrophytes, T. rubrum, M. canis*	Moazeni et al. (2021)
*Lippia origanoides*	Nanoemulsion	157–181	0.145–0.179	96.3	11.2–234.4 µg/mL	*T. mentagrophytes, T. tonsurans, E. floccosum, M. canis*	Kotwiski et al. (2024)
*Melaleuca alternifolia*	Nanoemulsion	160–220 nm	0.25	NA	0.25–1 μg/mL	*T. rubrum*	Flores et al. (2013)
	Nanocapsule	160–220 nm	0.25	NA	0.25–1 μg/mL	*T. rubrum*	Flores et al. (2013)
*Stenachaenium megapotamicum*	Nanoemulsion	210	0.369	77.6	5.18–41.5 µg/mL	*E. floccosum, T. rubrum*	Danielli, et al. (2013)
*Pinus sylvestris L.* essential oil (PSEO) + Voriconazole (VCZ)	Nanoemulsion	19.015 ± 0.110	0.146 ± 0.011	NA	80.33 ± 4.61 mm	*M. canis*	Al-Suwaytee et al. (2024)
*Lippia origanoides*	Nanoemulsion	157–181	0.145–0.179	96.3	11.2–234.4 µg/mL	*T. mentagrophytes, T. tonsurans, E. floccosum, M. canis*	Kotwiski et al. (2024)
Piperlongumine	Niosomal gel	93.23 ± 1.22 nm	0.289 ± 0.009	77.3 ± 2.04%	NA	*T. indotineae*	Haghani et al. (2025)
*Zataria multiflora* (ZM)	Nanostructured lipid carrier (NLC)	273.5 ± 4	0.33 ± 0.03	NA	0.004–0.0001 μg/mL	*T. rubrum, T. mentagrophytes, T. tonsurans, M. canis*	Fard et al. (2023)
Ozonated sunflower seed oil	Polymeric nanocapsules	150 (avg ~ 143 nm by DLS)	0.12	53	0.0011–0.0381 μg/mL	*T. rubrum, T. mentagrophytes, M. canis*	Dos Santos et al. (2023)
Copaiba oil	Solid lipid nanoparticle	126.06 ± 9.84	0.11 ± 0.04	NA	1.95 µg/mL	*T. rubrum, M. canis*	Svetlichny et al. (2015)
Copaiba oil + allantoin	Solid lipid nanoparticle	118.63 ± 8.89	0.07 ± 0.01	NA	1.95 µg/mL	*T. rubrum, M. canis*	Svetlichny et al. (2015)
*Satureja montana*	Beta-cyclodextrin complex	NA	NA	NA	20–100 µg/mL	*M. gypseum, M. canis, T. mentagrophytes, T. rubrum, T. tonsurans*	Haloci et al. (2014)
Oregano essential oil (OEO)	Nanoliposomes	77.46	0.302–0.479	79.55	1.5 μL/mL	*T. rubrum*	Aguilar-Pérez et al. (2021)
*Cinnamomum* ssp. (EOC)	Nanogel	176.0 ± 54.3		89.0 ± 4.0	400 μg/mL	*M. canis*	Carvalho et al. (2021)
*Syzygium aromaticum* (EOS)	Nanogel	263.0 ± 81.4	74.0 ± 3.0	74.0 ± 3.0	400 μg/mL	*M. canis*	Carvalho et al. (2021)
*Acanthospermum australe*	Silver nanoparticles	15 ± 3	0.270	NA	0.03–2 µg/mL	*E. floccosum, M. canis*	Mussin and Giusiano (2024)
*Achillea santolina*	Silver nanoparticles	21.9	NA	NA	128 μg/mL	*T. rubrum*	Abdallah et al. (2023)
*Taxandria fragrans*	Gold nanoparticles	NA	NA	NA	0.06–0.25 μg/mL	*T. interdigitale*	Carmo et al. (2021)
*Melaleuca alternifolia*	Gold nanoparticles	NA	NA	NA	0.12–0.50 μg/mL	*T. interdigitale*	Carmo et al. (2021)

^*^PDI polydispersity index

^**^ZOI zone of inhibition

It is not easy to say which type of nanoformulation of EO is most suitable for treating dermatophyte infections, but ultimately the choice will depend on the specific EOs or antifungal agent used and the location of the infection (e.g., skin vs. nail). However, lipid-based nanoparticles can easily overcome skin barriers to target the infection site through sustained release, making them the most promising nanocarriers for treating dermatophytes.

## Safety considerations of using essential oil

### Safety and ingredient security

EO nanoformulations showed potential as antimicrobial agents in the food and pharmaceutical industries, but their safety must be carefully assessed (Chaudhari et al. 2021a). Although EOs are generally considered safe, adverse effects such as skin irritation, redness, itching, burning sensations, and allergic contact dermatitis have been reported, especially at higher concentrations or in sensitive populations (Dermnetnz xxxx; Lakshmayya et al. 2023). Compounds such as Cinnamaldehyde (cinnamon), eugenol (clove), and citral (lemongrass) are known irritants and allergens (Tammannavar et al. 2013; Heydorn et al. 2003). Therefore, EOs should be diluted, especially for children and older adults, to avoid skin irritation, and patch testing is essential to identify potential side effects. Proper consultation with healthcare providers must be recommended, especially for pregnant women, those with other medical ailments, and those on medication and specific skin conditions, before using EOs (Sarkic And Stappen 2018).

### Nano-specific safety and toxicological considerations

Encapsulating EOs in nanocarriers can significantly change their toxicity profile, biodistribution, and biological interactions. However, by releasing EOs in a controlled and sustained manner can reduce irritation-related side effects. Dose-dependent toxicity has been reported when the concentration of EOs increased within the nanocarrier (Bilia et al. 2014). The acute toxicity of EOs and their nanoemulsions is typically assessed using LD_50_ values in mammalian models, with considerable variation observed among different EOs such as *Petroselinum crispum, Pimpinella anisum*, and *Illicium verum* (Chaudhari et al. 2021b; Das et al. 2021; Dwivedy et al. 2018). These findings emphasize the significance of comprehensive in vivo toxicological evaluations during formulation development.

Regulatory authorities, including the U.S. Food and Drug Administration (FDA) and the European Medicines Agency (EMA), require additional safety assessments for nanomaterials. These include physicochemical characterization, nanoparticle dispersion stability, degradation behaviour, cellular reactivity, and pharmacokinetic and pharmacodynamic profiling (Maurya et al. 2021; Prakash et al. 2024). Despite these guidelines, there are still limited standardized regulatory pathways for EO nanoformulations, which restrict their clinical translation. Therefore, EO nanoformulations must not be used in clinical practice without the necessary regulatory approval and medical supervision.

### Biocompatibility and skin compatibility

Although EO nanoformulations enhance the delivery of bioactive compounds, dermal biocompatibility is essential. Allergic reactions associated with EOs and stability issues with stabilizers and lipids remain ongoing concerns. Additionally, nanoparticle aggregation can influence EO delivery and skin permeation (Ashraf et al. 2023). Lipid nanoparticles (LNPs) and nanostructured lipid carriers (NLCs) have demonstrated improved biocompatibility and enhanced skin hydration compared to conventional creams, with no evidence of toxicity or structural skin damage (Stefanov And Andonova 2021; Ashfaq et al. 2023). Cyclodextrins further enhance EO biocompatibility by improving solubility and stability without posing toxicity risks, as observed with *Helichrysum italicum* oil (Jakupović et al. 2023). Careful formulation design and thorough testing are essential to ensure that EO nanoformulations are safe and effective for dermal applications, thereby minimizing adverse reactions while maximizing therapeutic benefits (Naahidi et al. 2013).

### Storage stability and product integrity

Appropriate storage conditions are essential for maintaining the stability and efficacy of EO nanoformulations. Factors such as temperature, oxygen exposure, light, and moisture significantly influence shelf life and performance. Maintaining storage temperatures between 4 and 15 °C can inhibit degradation and microbial growth (Hategekimana et al. 2015; George et al. 2019). Opaque containers protect against photodegradation, while anoxic packaging limits oxidation (Sivakumar et al. 2021). The use of glass containers, silica gel for moisture control, multilayer polymer systems, and nitrogen pressurization during filling further enhances formulation stability and shelf life (Hategekimana et al. 2015; Asare et al. 2022).

### Compatibility with drug matrix and dosage control

Compatibility between EO nanoformulations and pharmaceutical excipients is crucial to ensure controlled release, prevent nanoparticle aggregations, and maintain product uniformity (Manuja et al. 2021). Stability under different pH levels, temperatures, and ionic strengths, uniform appearance, color, and consistency of the nanoemulsion are essential for product acceptability. By adding odor-masking agents to reduce unpleasant taste in orally administered formulations is good to patient compliance (Preeti et al. 2023). Dosage varies depending on application and concentration, typically ranging from 0.1 to 2% for cosmetic use (Bilal et al. 2020) and 5–15% for muscular applications when diluted in carrier oils (Paul et al. 2020).

### Drug interactions and photosensitization

EO nanoformulations may influence pharmacokinetics and pharmacodynamics due to their lipophilic nature and interactions with metabolic enzymes such as cytochrome P450 (Yingprasert et al. 2015; Sadgrove et al. 2021). Interactions have been reported with anticoagulants, CNS depressants, antidiabetic agents, and chemotherapeutic drugs (Shamsi et al. 2017; Fekrazad, et al. 2015; Bernardo and Valentão 2024). Certain EOs, particularly citrus oils, can cause photosensitivity, leading to phototoxic reactions upon UV exposure (Ailioaie et al. 2021). Additionally, nanoformulated photosensitizers may generate singlet oxygen under light activation, necessitating careful evaluation of photoreactivity and biocompatibility (Mesquita et al. 2018).

## Challenges and future perspectives

EOs have a clear advantage because of their multi-target mechanisms of action. Unlike single-target synthetic drugs, some EOs like Tea Tree and Lemongrass act simultaneously in multiple points. Nanoformulations, such as liposomes and nanoemulsions, are effective delivery systems that address the inherent limitations of EOs, specifically their high volatility and poor skin penetration. The EOs nanoformulations offer a novel approach to treating fungal infections, especially those caused by dermatophyte species such as *T. mentagrophytes*, *M. canis,* and *E. floccosum*. However, their topical application is limited by several challenges, such as instability under environmental stress, volatility, and hydrophobicity (Abd Rashed et al. 2021). Furthermore, the effectiveness of EO nanoformulations can be compromised by factors such as EO composition, formulation, and storage conditions (Nieto Marín et al. 2025). Safety and skin irritation may be reported due to dose-dependent toxicity by the EO compound when delivered via nanocarriers (Bilia et al. 2014).

Despite these limitations, the prospects for treating fungal infections with EO-nanoformulations are up-and-coming. Biogenic synthesis of nanoparticles using plant extracts or biodegradable materials provides a safer, more suitable approach for treating fungal infections (Moosavy et al. 2023). In addition, synergistic combinations of metal nanoparticles or conventional antifungal drugs with EOs may help to overcome resistance to treat dermatophytosis (Abdallah et al. 2023). Future work should focus on in vivo pharmacokinetic techniques, advanced transdermal delivery strategies such as microneedle-assisted or hydrogel-based nanocomposites for targeted delivery, and careful evaluation of the toxicity profile in clinical trials.

## Conclusion

Reappearance of lesions may occur with conventional antifungal drugs due to adverse effects, the development of microbial resistance, and the prolonged treatment required for dermatophyte infections. In this context, EOs showed a great potential as a novel treatment option. Numerous studies support the use of EOs in traditional medicine for the treatment of various human pathogens, including fungal infections. EO nanoformulations further improve topical applications for treating dermatophyte infections by increasing the bioavailability of active components in the skin layer, thereby enhancing antifungal activity. Moreover, nanoformulations allow reduced concentrations for effective treatment, minimizing potential side effects. The safety of EO nanoformulations depends on the materials used in the formulation and the bioactive components incorporated. A comparative analysis of the delivery systems discussed in this review shows that nanoemulsions (NEs), liposomes, and solid lipid nanoparticles (SLNs) are the most efficient platforms for delivering EOs in antifungal therapy. While NEs enhance solubility and skin penetration, liposomes and SLNs offer advantages in biocompatibility and controlled release, respectively. By transitioning from free EOs to nanoencapsulated systems, it is possible to achieve higher bioavailability and prolonged anti-dermatophytic activity.

Established procedures exist to evaluate formulation safety; however, comprehensive clinical trials are essential before widespread adoption. Furthermore, interactions with other drugs must be evaluated, as well as possible adverse effects. Despite these considerations, the protective advantages of nanoformulations make them promising in the pharmaceutical industry, with several applications reported against fungi, including dermatophytes. EO nanoformulations hold promise, but over-reliance on these natural products should be avoided, and physician guidance remains indispensable to antifungal therapy. These advancements represent a pivotal step toward developing standardized, natural-based alternatives to conventional antifungals, effectively addressing limitations such as drug toxicity and the rapid degradation of bioactive volatiles.

## Data Availability

Data sharing is not applicable to this article.
